# ssDNA Aptamer Specifically Targets and Selectively Delivers Cytotoxic Drug Doxorubicin to HepG2 Cells

**DOI:** 10.1371/journal.pone.0147674

**Published:** 2016-01-25

**Authors:** Ge Yu, Huan Li, Shuanghui Yang, Jianguo Wen, Junqi Niu, Youli Zu

**Affiliations:** 1 Department of Hepatology, The First Hospital of Jilin University, Changchun, Jilin Province, China; 2 Department of Pathology and Genomic Medicine, Houston Methodist Hospital, Houston, Texas, United States of America; 3 Xiangya Hospital of Central South University, Changsha, China; Consiglio Nazionale delle Ricerche (CNR), ITALY

## Abstract

Hepatocellular carcinoma (HCC) is the third leading cause of death due to cancer worldwide with over 500,000 people affected annually. Although chemotherapy has been widely used to treat patients with HCC, alternate modalities to specifically deliver therapeutic cargos to cancer cells have been sought in recent years due to the severe side effects of chemotherapy. In this respect, aptamer-based tumor targeted drug delivery has emerged as a promising approach to increase the efficacy of chemotherapy and reduce or eliminate drug toxicity. In this study, we developed a new HepG2-specific aptamer (HCA#3) by a procedure known as systematic evolution of ligands by exponential enrichment (SELEX) and exploited its role as a targeting ligand to deliver doxorubicin (Dox) to HepG2 cells in vitro. The selected 76-base nucleotide aptamer preferentially bound to HepG2 hepatocellular carcinoma cells but not to control cells. The aptamer HCA#3 was modified with paired CG repeats at the 5′-end to carry and deliver a high payload of intercalated Dox molecules at the CG sites. Four Dox molecules (mol/mol) were fully intercalated in each conjugate aptamer-Dox (ApDC) molecule. Biostability analysis showed that the ApDC molecules are stable in serum. Functional analysis showed that ApDC specifically targeted and released Dox within HepG2 cells but not in control cells, and treatment with HCA#3 ApDC induced HepG2 cell apoptosis but had minimal effect on control cells. Our study demonstrated that HCA#3 ApDC is a promising aptamer-targeted therapeutic that can specifically deliver and release a high doxorubicin payload in HCC cells.

## Introduction

Hepatocellular carcinoma (HCC) is the fifth most common malignant tumor and the third most common cause of cancer-related mortality worldwide, with 50% of cases occurring in China [1–3]. Patients diagnosed with advanced HCC have survival rates of less than 5 years, considerably less than those with early-stage disease. Only 20% of the patients diagnosed with HCC are amenable to curative therapy such as liver transplantation [LT], surgical resection [SR] or ablative therapies. Additionally, HCC often recurs even after curative therapy and the survival rate of patients with advanced stage HCC remains poor [4–7]. Locoregional therapies such as radiofrequency ablation (RFA), percutaneous ethanol injection (PEI), microwave coagulation therapy and transcatheter arterial chemoembolization (TACE) play a key role in the management of unresectable HCC tumors. The previous non-surgical treatments for HCC have been significantly improved in the last few decades and have increased the patient survival in several cases [5, 6, 8–11]. Currently, molecular-targeted agents such as sorafenib have emerged as promising drugs for advanced HCC; however, they extend the overall survival by 2–3 months only. Moreover, side effects such as diarrhea, weight loss, hand-foot syndrome, and hypophosphatemia occur more frequently in patients treated with sorafenib [12].

Aptamers, or “chemical antibodies”, are a new class of small multifunctional ligands comprising short single-stranded oligonucleotides about 60–80 bases in length with high affinity and specificity for their targets [13]. Aptamers are developed from RNA or ssDNA libraries via an experimental directed process referred to as Systematic Evolution of Ligands by Exponential enrichment (SELEX) [14, 15]. Compared to protein antibodies, aptamers are synthesized easily and exhibit negligible immunogenicity in vivo [16–18]. Additionally, aptamers have been developed as effective targeted drug delivery systems to reduce or eliminate the severe side effects of chemotherapy [19] as well as molecular recognition probes for cancer detection [20–23]. To this effect, potent cytotoxic drugs have been conjugated directly to judiciously chosen aptamers via covalent or non-covalent interactions [24]. The ensuing aptamer-drug conjugates exhibited enhanced therapeutic efficacy compared to the free drug and reduced nonspecific toxicity [24].

HepG2 is a perpetual line of human liver carcinoma cells often used as an HCC model. HepG2 was derived from the liver tissue of a 15-year-old Caucasian male who had a well-differentiated hepatocellular carcinoma. In this study, we developed a HepG2-specific aptamer, HCA#3, which selectively binds to HepG2 but not to the control cells SK-HEP-1 derived from the ascetic fluid of a patient with liver adenocarcinoma. Furthermore, we prepared the HCA#3 aptamer-drug conjugate (ApDC) with doxorubicin (Dox) and demonstrated that the synthetic ApDC specifically induced apoptosis in HepG2 cells but had minimal apoptotic effect on the control cells.

## Materials and Methods

### Cell Lines and Reagents

HepG2 and SK-HEP-1 (Hepatocellular carcinoma) cell lines were cultured in Eagle’s Minimum Essential Medium (EMEM) medium. CA-46 (Burkitt’s lymphoma), SU-DHL-1 (Anaplastic Large Cell Lymphoma), and KMH2 (Hodgkin lymphoma) cell lines were cultured in RPMI-1640 medium (GIBCO, Grand Island, NY, USA); PANC-28 (Pancreatic Cancer), Hey (Ovarian Cancer), DU145, 22RV1, LNCAP, and PC3 (Prostate cancer) cell lines were cultured in Dulbecco's Minimum Essential Medium (DMEM) supplemented with 10% heat-inactivated Fetal Bovine Serum (FBS) (GIBCO) and 100 IU/mL penicillin-streptomycin. All the cell lines were obtained from the American Type Culture Collection (ATCC, Manassas, VA, USA).

Dulbecco’s Phosphate-Buffered Saline (DPBS; Sigma, St. Louis, MO, USA) enriched with 4.5 g/L glucose and 5 mM MgCl_2_ was used as washing buffer during selection and flow cytometry assays. The binding buffer, comprising washing buffer with 1 mg/mL Bovine Serum Albumin (BSA; Fisher, Waltham, MA, USA) and 0.1 mg/mL t-RNA, was used to reduce nonspecific background binding.

### SELEX Primers and DNA Library

The ssDNA library for SELEX contained a random core of a 40mer oligonucleotide flanked by 18mer primer sequences binding at both ends as previously reported [25]. A biotinylated reverse primer 5’- ACTAAGCCACCGTGTCCA -3’ was used to generate single-stranded DNA, and a fluorophore Cy3-labeled forward primer 5’-ATCCAGAGTGACGCAGCA -3’ was used to monitor the progress of aptamer selection. The aptamer pools were amplified by PCR with Taq polymerase. PCR reagents were purchased from Takara Bio (Mountain View, CA, USA).

### Cell-Based SELEX

Selection was performed using HepG2 cells in a 100-mm × 20-mm culture dish (over 5 million cells). After washing twice with buffer, cells were incubated with a DNA library, and rapidly cooled on ice after heating at 95°C for 5 min. To increase the stringency of selection, a reduced number of HepG2 cells were used in the subsequent 3 rounds of selection. After incubation, cells were treated thrice with washing buffer and detached using a cell scraper. The cell suspension was heated at 95°C for 10 min and the suspended DNA was recovered by centrifugation at 13,100 x g for 5 min. The eluted DNA was amplified by PCR; the PCR conditions were optimized with *Taq* polymerase to yield a clear, single band after each SELEX round. High-affinity streptavidin-sepharose beads were used to capture the biotinylated anti-sense strands, and the sense strands with the fluorophore were eluted with 200 mM NaOH. The resulting ssDNA was used for the next round and the process was repeated iteratively until significant affinity towards the target HepG2 cells was observed by flow cytometry. The stringency of selection was also increased by reducing the incubation time and the concentration of the aptamer pool used for selection as well as by increasing the washing time and volume [25]. The selection progress was tested by flow cytometry using Cy3-labeled primers to generate a fluorescent-labeled pool with HepG2 cells. The selection was stopped when no further progress was observed after 4 rounds of selection.

### Sequencing of DNA Aptamers

The final ssDNA pool was PCR-amplified with unmodified primers, purified with a ChargeSwitch PCR Clean-Up Kit (Life Technologies), and submitted for next-generation sequencing (LC Sciences LLC, Houston, TX, USA) [26].

### Flow Cytometric Analysis

The aptamer pools were tested using 100 nM of the enriched pool to monitor SELEX. HepG2 and control cells (5 x 10^5^) were treated with washing buffer, incubated in 100 μL binding buffer for 30 min, washed again with buffer (1 ml), and then re-suspended in 300 μL DPBS. Ten thousand cells were counted on a BD Fortessa flow cytometer (Becton Dickinson Immunocytometry Systems, Franklin Lakes, NJ, USA). The fluorescence readouts depend on the affinity of the fluorophore-labeled pools for HepG2 cells. The aptamers were prepared, and the affinities of Cy3-labeled aptamers for different types of cancer cells were recorded. Apparent dissociation constants K_d_ were measured by flow cytometry after serial dilution of the aptamers.

### Fluorescence Microscopy

Imaging was performed with a fluorescence microscope (Olympus America, Melville, NY). HepG2 and SK-HEP-1 (control) cells were incubated with 100 nM of Cy3-labeled aptamer HCA#3 for 1 h, and then treated twice with washing buffer (2 x) in 100 μL medium for imaging.

### Determination of HepG2-specific aptamer biostability *in vitro*

DNA aptamers (1 μg) were incubated with human serum for 0, 1, 2, 4, 8, and 24 h. These samples were subjected to the standard phenol-chloroform DNA extraction protocol and the recovered aptamers were visualized on a 3% agarose gel [27].

### Doxorubicin uptake by the HepG2-targeting aptamer

Dox was purchased from Thermo Fisher Scientific. Because Dox preferentially intercalates at paired 5’-GC-3’ or 5’-CG-3’ sites [28], CG repeats were added to the 5’ end of the aptamer HCA#3 to synthesize the modified aptamer HCA#3 -CG. To determine the optimal molar ratio of aptamer to Dox, aptamer HCA#3 was heated at 95°C for 5 min, immediately cooled on ice for 15 min, and then incubated with a fixed concentration of Dox (final Dox concentration 0.5 μmol/L) in DPBS at various aptamer/Dox ratios ranging from 1:40 to 1:4. After 1 h, the fluorescence (λ_ex_ = 488 nmol/L, λ_em_ = 590 nmol/L was measured with a Synergy4 analyzer (BioTek, Winooski, VT). The optimal molar ratio of aptamer to Dox (1:4) [29] was determined as the minimum ratio at which the fluorescence of Dox was quenched.

### Determination of HCA#3 ApDC biostability in serum-containing medium

The stability of ApDC in culture medium containing 1 and 5% FBS was studied as previously described [30].

### Cellular uptake of Doxorubicin

The cellular uptake of Dox was evaluated by fluorescence microscopy (Olympus America, Melville, NY). Cells were grown in 12-well plates for 24 h, and then incubated with 2 μM Dox or ApDC (final equivalent concentration of aptamer was 0.5 μM and DOX was 2 μM) complex for 2 h at 37°C. After washing with DPBS, 2 ml of complete medium was added into each well before fluorescence microscopy analysis.

### *In vitro* annexin V cytotoxicity assays

To evaluate the cytotoxicity of ApDC or Dox against HepG2 and control cells, cell lines were grown in 12-well plates, and then incubated in complete medium containing 10% FBS with ApDC, Dox, or aptamer at specific concentrations for 2 h at 37°C. Cells were washed with DPBS, and cultured for 48 h before the apoptosis assay was performed as previously reported [19].

### Western blotting

The cytotoxicity of ApDC or Dox against HepG2 and control cells was assessed by Western blotting using anti-cleaved caspase-3 and anti-cleaved PARP antibody, as previously reported [19].

### Statistics

Standard deviation values in the binding affinity graphs are depicted with error bars; the average readings from two different experiments in duplicate were used. The graphs were plotted using MS-Excel 2010, and the integral Solver tool pack was used for data analysis.

## Results

### Aptamer selection

**Fig 1:**
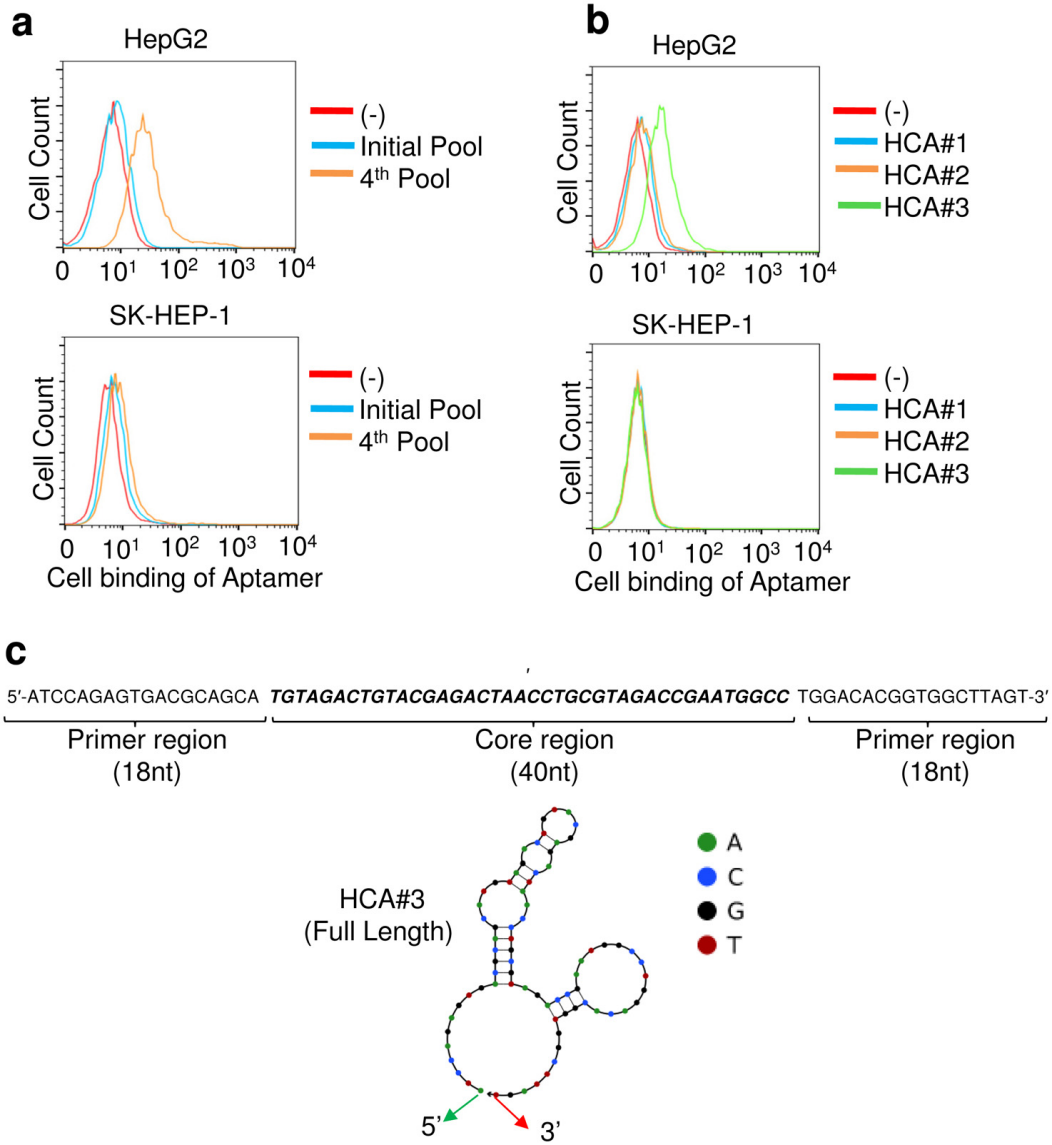
Selection, identification, and optimization of aptamers specific for HCC tumor cells. (a) Binding assay of selected pool for HepG2 and SK-HEP-1 cells. Flow cytometry assay to monitor the binding of selected pool with HepG2 cells (target cells) and SK-HEP-1 cells (control cells). The blue curves represent the background binding of the unselected DNA library. An increase in the binding capacity of the pool for HepG2 cells during the selection process was observed as opposed to small changes for the control SK-HEP-1 cells. (b) Representative aptamer sequences from each group, aptamers #1, #2, and #3, were synthesized and conjugated with a Cy3 fluorochrome reporter. Synthetic aptamers were incubated with cultured HCC (HepG2) and control cells (SK-HEP-1) and the resulting cell binding was quantified by flow cytometry. HCA#3 showed the highest binding affinity and specificity. (c) Individual aptamer sequence HCA#3; predicted 2D structure of aptamer HCA#3 including 5’- and 3’- primers, and the central core.

The SELEX process was monitored by flow cytometry to test the binding affinity of the enriched aptamer pool for HepG2 cells. The SELEX scheme applied for the aptamer selection is depicted in S1 Fig. The upper panel in Fig 1a depicts the affinity of the pool for HepG2 cells after 4 rounds of SELEX and the lower panel depicts the affinity of the pool for SK-HEP-1 control cells. The SK-HEP-1 cell line was derived from the ascetic fluid of a patient with liver adenocarcinoma. In contrast to HepG2 cells, the SK HEP-1 cell line does not exhibit hepatocyte cell properties [31] despite the location where the tumor was derived. Thus, we used SK-HEP-1 as the control cell line. The results showed that the aptamer pool after 4 rounds of enrichment is highly enriched for HepG2 cells but has no affinity for the control cells and the ssDNA pool was then submitted for next-generation sequencing (Fig 1a).

### Aptamer HCA#3 selectively recognized HepG2 cells

**Fig 2:**
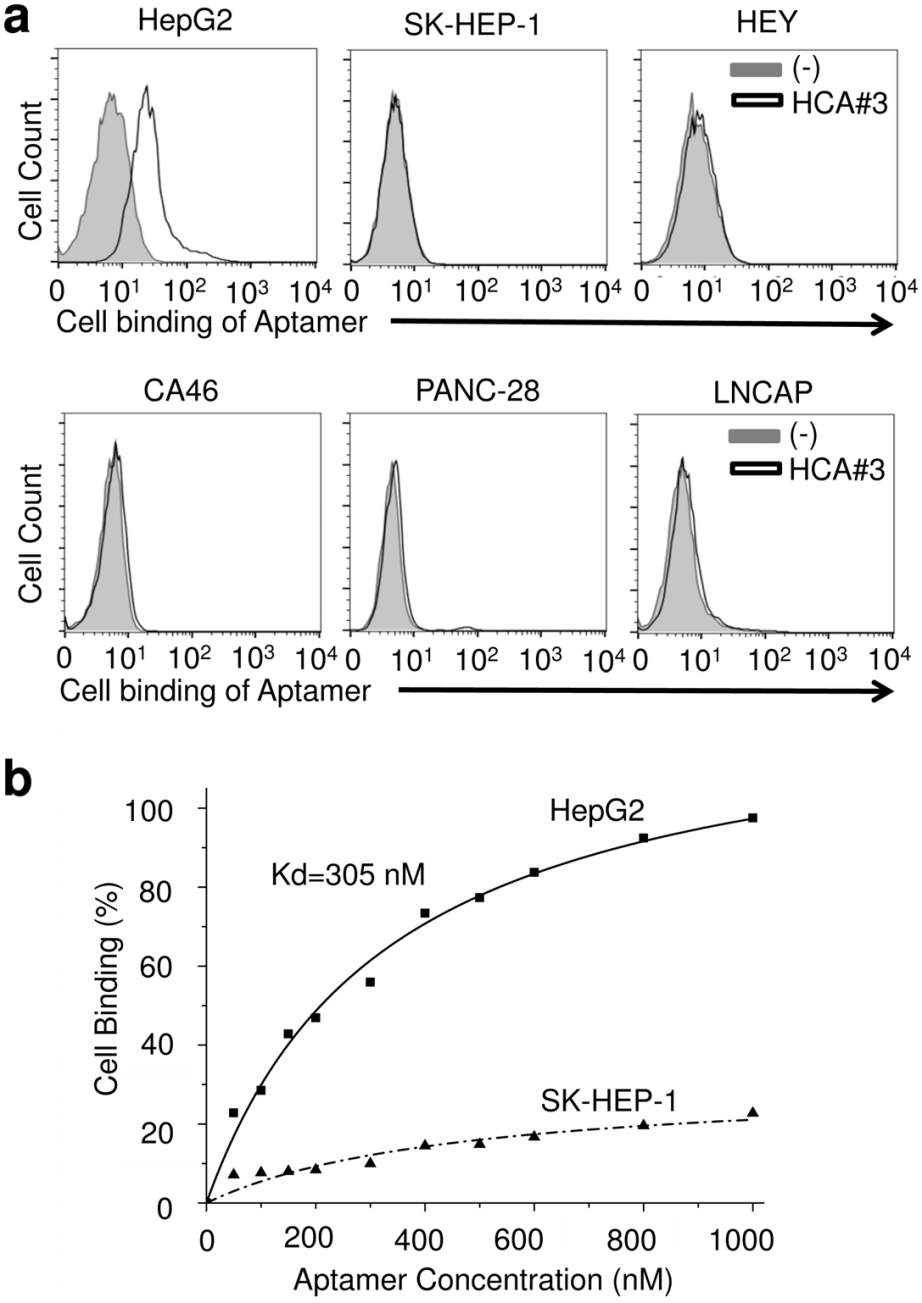
Aptamer HCA#3 specifically recognizes HepG2 cells. (a) HepG2 cells were stained with aptamer HCA#3 and cell binding was determined by flow cytometry. HCC (SK-HEP-1), pancreatic (PANC-28), B-cell lymphoma (CA46), ovarian (Hey) and prostate cancer (LNCAP) cell lines were examined by flow cytometry under the same conditions. (b) Plot with apparent dissociation constant K_d_ measurements for aptamer HCA#3 with HepG2 and SK-HEP-1 cell lines.

**Fig 3:**
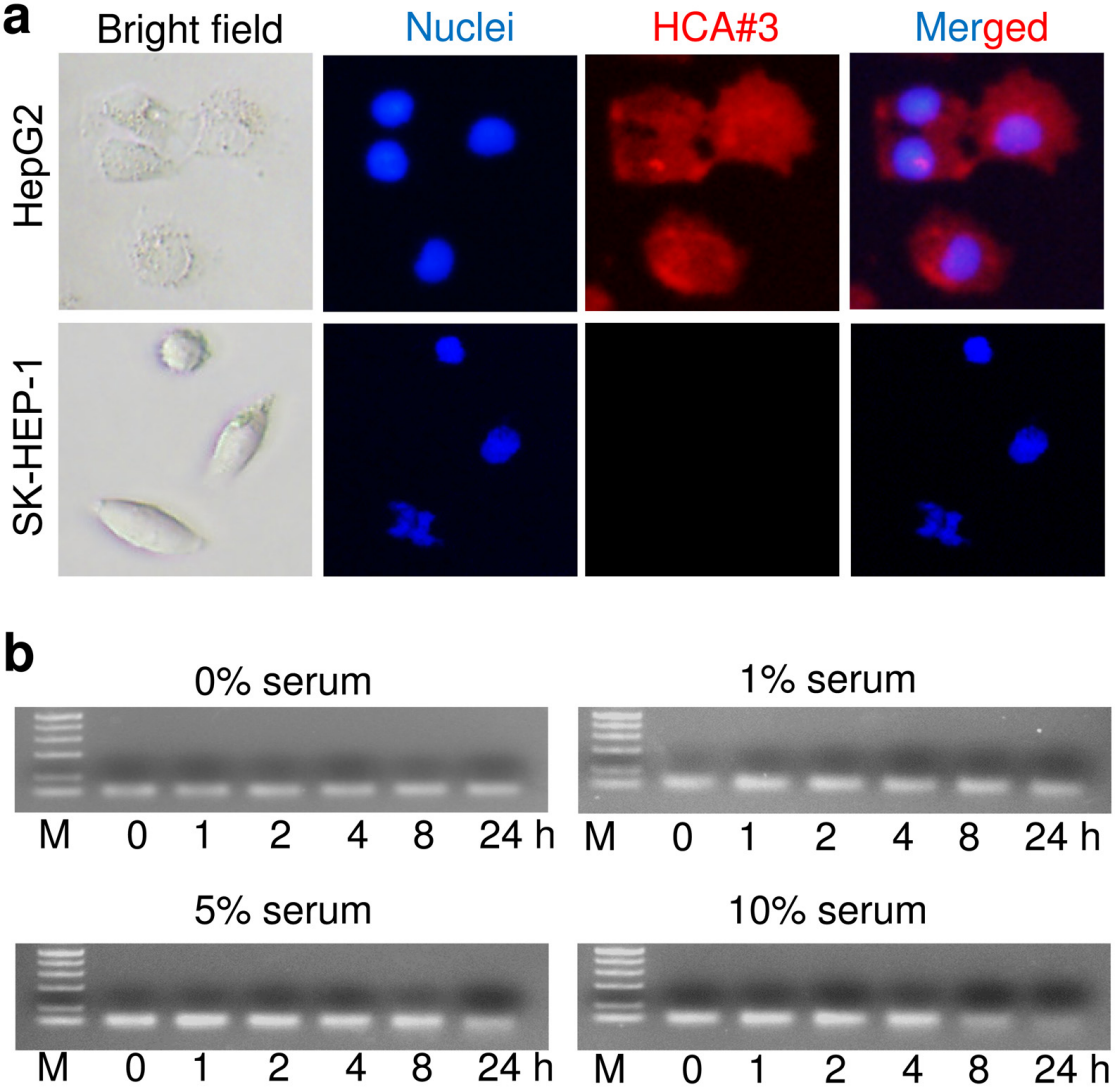
Binding assay and biostability assay of HCA#3. (a) Binding assay of HCA#3 with HepG2 and control cells. Fluorescence imaging of cells bound to aptamer HCA#3 labeled with Cy3. (b) Biostability of aptamers; HCA#3 aptamers recovered from incubation with complete medium containing 0, 1, 5, or 10% FBS were visualized on 3% agarose gel.

Among the enriched sequences, HCA#3 showed the highest selectivity (Fig 1b) and hence was selected for further analysis. The secondary structure of the aptamer HCA#3 was predicated by using the Mfold software (Fig 1c) [32, 33]. The sequences and secondary structures of the other two aptamers, namely HCA#1 and HCA#2, are shown in S2 Fig. To confirm the specificity of the HCA#3 aptamer, multiple cultured cancer cell lines were stained with HCA#3. As shown in Fig 2a, the aptamer specifically recognized HepG2 cells but not the control cells. A complete list with the results from the aptamer binding studies for 12 cell lines is presented in Table 1 [34]. In addition, the cell-binding affinity of the aptamers was analyzed by applying serial dilution. Flow cytometry assays revealed that aptamer HCA#3 had a high binding affinity for HepG2 and an apparent K_d_ = 305 nM (Fig 2b). Specific binding of the selected HCA#3 to the target cells was further confirmed by fluorescence imaging (Fig 3a). After incubation with the Cy3-labeled aptamer pool, the HepG2 cells displayed bright fluorescence whereas the signal from the SK-HEP-1 cells was weak. Furthermore, to determine the minimal region required for selective binding, HCA#3 was truncated into two different shorter aptamers HCA#3-S1 and HCA#3-S2, and their affinities were determined by flow cytometry (S3a and S3b Fig). Because the affinities of the two truncated aptamers were lower than that of the parental aptamer HCA#3, we used the full length HCA#3 for our studies.

**Table 1:** Summary of aptamer HCA#3 binding affinities for cancer cell lines.

Cell Line	Cancer	HCA#3 Aptamer
HepG2	Hepatocellular carcinoma	+++
SK-HEP-1	Hepatocellular carcinoma	-
PANC-28	Pancreatic Cancer	-
HEY	Ovarian Cancer	-
PC3	Prostate cancer	-
22RV1	Prostate cancer	-
LNCAP	Prostate cancer	-
DU145	Prostate cancer	-
CA46	Burkitt’s lymphoma	-
SU-DHL-1	Anaplastic Large Cell Lymphoma	-
KMH2	HodgKin lymphoma	-

The percentage of the cells with fluorescence above the set threshold was used to evaluate the binding capacity of the aptamer to the cells. 0, 10%; +, 10–35%; ++, 35–60%; +++, 60–85%; ++++, 85%.

### Biostability of ssDNA Aptamer

Susceptibility of aptamers to nucleases is one of the main challenges to be addressed for their use in vivo as they are required to be stable under physiologic conditions. HCA#3 aptamers were incubated with FBS to test their biostability. After incubating HCA#3 in complete medium containing 0, 1, 5 or 10% FBS for 0, 1, 2, 4, 8, and 24 h at 37°C, the aptamers were subsequently examined on 3% agarose gel. As shown in Fig 3b, HCA#3 had minimal degradation after incubation with serum for 0–24 h.

### Formation of ApDCs

**Fig 4:**
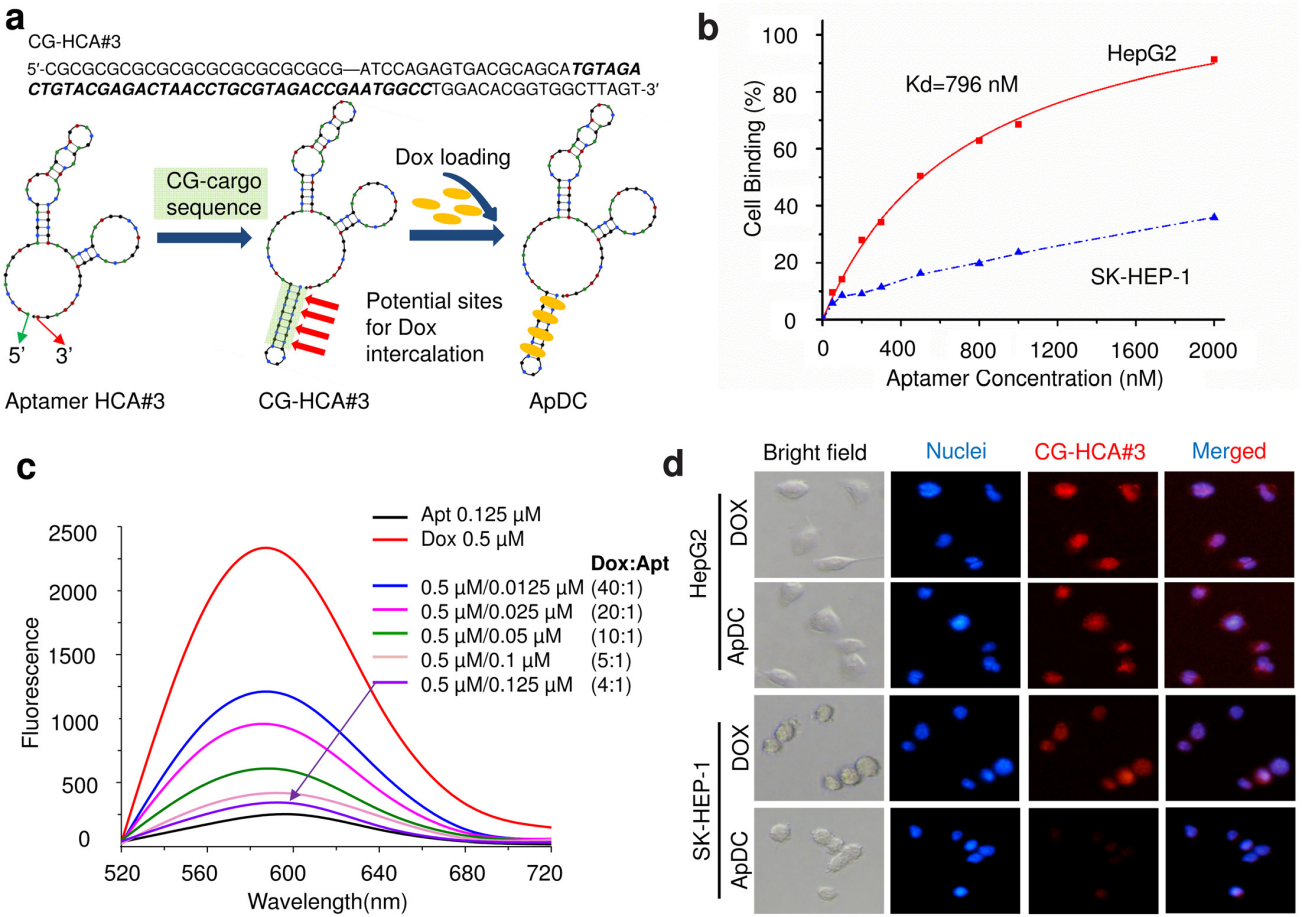
Formation of HCA#3 ApDC. (a) Schematic diagram of ApDC intercalation. (b) Plot with apparent dissociation constant K_d_ measurements for CG-HCA#3 with HepG2 and SK-HEP-1 cell lines. (c) Fluorescence spectra of the ApDC uptake experiment. Doxorubicin solution mixed with increasing amounts of HCA#3. (d) Dox uptake by HepG2 and control cells. Fluorescence microscopy images of HepG2 and SK-HEP-1 cells after incubation with free Dox or ApDC for 2 h.

For drug delivery, aptamer HCA#3 with CG-cargo was synthesized to carry a high payload of intercalated Dox molecules (Fig 4a) [28]. Importantly, HCA#3 aptamers with CG-cargo still retain the selective affinity for HepG2 cells (Fig 4b). Subsequently, Dox was conjugated with aptamer HCA#3 for selective delivery to HepG2 cells. We developed ApDC based on the fact that Dox intercalates in DNA. To evaluate the drug-loading capacity of HCA#3 ApDCs, a fixed concentration of Dox was mixed with HCA#3 aptamer at increasing molar ratios and the mixtures were analyzed by fluorescence spectroscopy. As shown in Fig 4c, the fluorescence of Dox decreased with increasing concentration of aptamer. The fluorescence of Dox was almost completely quenched when the Aptamer/Dox molar ratio gradually reached 0.25, which suggested that one molecule of HCA#3 ApDC can conjugate at least 4 molecules Dox. Notably, HCA#3 ApDC is stable in serum-containing medium and only 4 and 6.8% Dox were released from ApDC after incubation for two hours in medium containing 1 and 5% FBS, respectively (Table 2).

**Table 2:** Dox release from ApDC in serum-containing media.

Time	Dox released (%) (1% serum)	Dox released (%) (5% serum)
0 h	0.0	0.0
0.5 h	1.3	2.6
1 h	1.9	3.3
2h	4.0	6.8
24 h	19.1	21.3

Dox release percentage from ApDC after incubation with either 1% or 5% serum-containing medium at 37°C.

### Aptamers as vehicles for selective delivery of Dox to HepG2 cells

The adverse effect of Dox is mainly due to the nonselective uptake by both cancer and normal cells. When Dox is incorporated into ApDCs, however, the conjugate preferentially binds to HepG2 cells. To test this postulate, HepG2 and control cells were incubated with free Dox or ApDCs and analyzed by fluorescent microscopy. As shown in Fig 4d, the red fluorescence from Dox is comparable in the two cell lines treated with free Dox, suggesting a non-selective uptake of Dox. However, in the ApDC-treated group, the red fluorescence from Dox in HepG2 cells is significantly higher than that in SK-HEP-1 cells (Fig 4d), a finding strongly suggesting that ApDC delivers Dox to HepG2 cells selectively.

**Fig 5:**
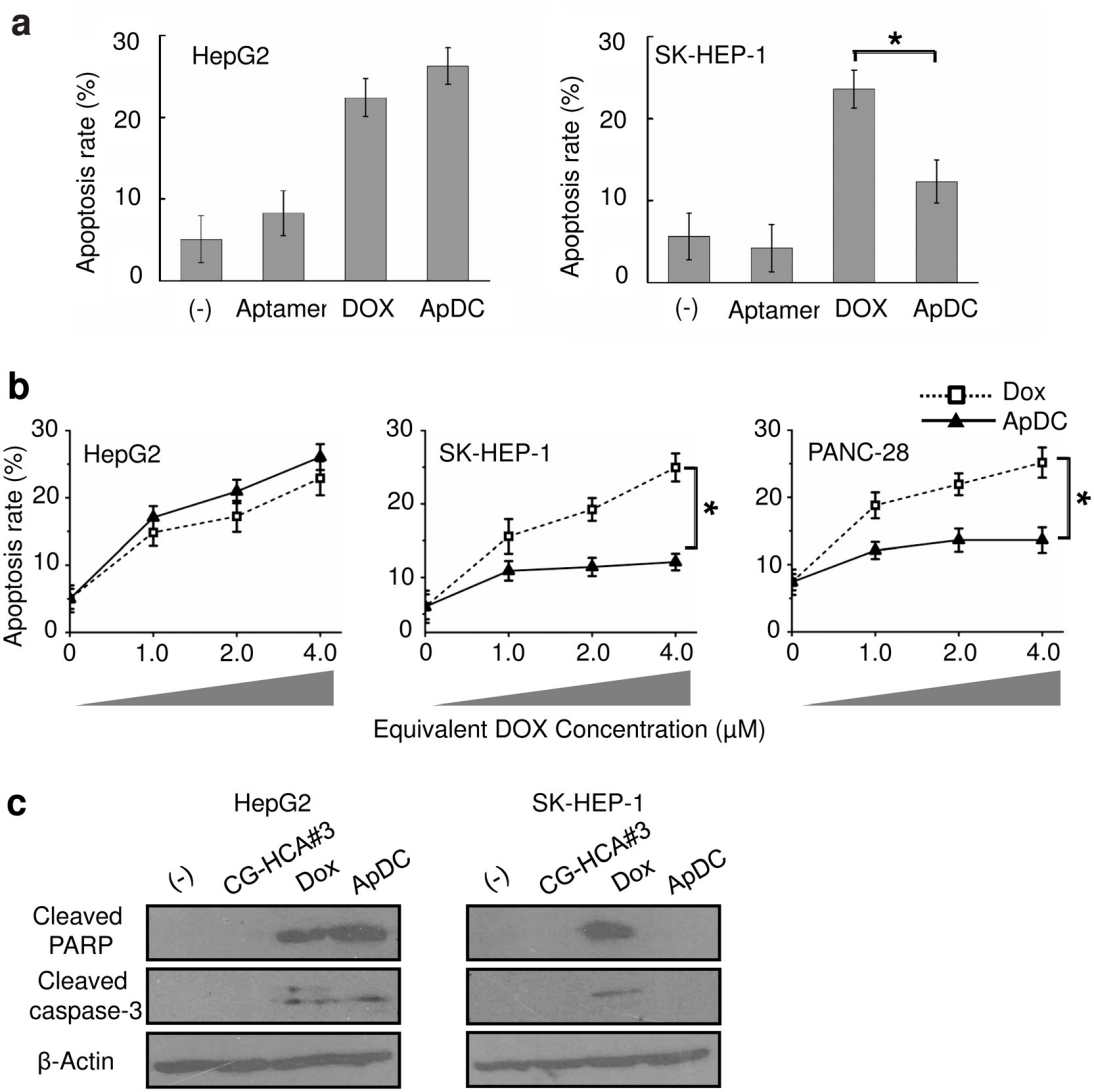
ApDC specifically delivered Dox into HepG2 cells. (a) Cytotoxicity assays of Dox and ApDC. The HepG2 and the SK-HEP-1 cells were treated with aptamer, free Dox (2 μM), or ApDC (final equivalent concentration of aptamer was 0.5 μM and DOX was 2 μM). After incubation for 48 h, cell viability was evaluated by the annexin V apoptosis assay (mean ± SD, n = 3). The star indicates a statistically significant difference between Dox and the ApDC groups (p<0.05). (b) HepG2, SK-HEP-1, and PANC-28 cells were treated with increasing concentrations of Dox and ApDC, and cell apoptosis was measured after 48 h. (c) HepG2 and SK-HEP-1 cells were treated with aptamer, free Dox (2 μM), or ApDC (final equivalent concentration of aptamer was 0.5 μM and DOX was 2 μM). After incubation for 48 h, Western blotting analysis was performed on HepG2 and SK-HEP-1 cells using antibodies specific for cleaved-PARP and cleaved-caspase-3. β-Actin was used as a loading control.

To examine whether the selective delivery of ApDC to HepG2 cells would result in targeted cytotoxicity, we compared the cytotoxic effects of free Dox and ApDC on both HepG2 and control cells *in vitro* by performing the annexin V assay. The data showed that free Dox had similar cytotoxicity effects on both cancer cell lines, whereas ApDC induced significantly reduced cytotoxicity in the control cell line (Fig 5a). Moreover, ApDC-induced cytotoxicity in HepG2 cells exhibited a dose-dependent effect (Fig 5b). This ApDC induced apoptosis was further confirmed with Western blot using anti-cleaved caspase-3 and anti-cleaved PARP antibody. Results showed that HCA#3 ApDC selectively induced up-regulated expression of cleaved caspase-3 and cleaved-PARP in HepG2 cells but not in the control cells (Fig 5c).

## Discussion

Aptamer drug conjugation has emerged as an advantageous approach to deliver cytotoxic drugs to cancer cells and inhibit their proliferation with minimal off-target effects to the healthy cells. To this end, aptamer-mediated targeted therapies have been recently explored with some conjugates currently undergoing clinical trials. In a few recent studies, Dox, a chemotherapeutic used widely in the treatment of various cancers, was conjugated covalently or non-covalently to aptamers at preferred paired GC or CG steps. The ensuing aptamer-drug conjugates enhanced the therapeutic efficacy of the drugs and reduced considerably the side effects of the treatment. For example, the aptamer-DOX conjugates EpDT3-Dox, HER2-Dox, MUC1-Dox and PSMA-Dox were successfully used in the treatment of retinoblastoma [35], breast [36], lung [37], and prostate [29] cancers, respectively. Moreover, Xing et al. developed AS1411 aptamer-functionalized liposomes containing doxorubicin as a payload for targeted breast cancer therapy [38]. Currently, several aptamer-based anticancer therapeutics are undergoing pre-clinical and clinical trials [20]. For example, the aptamers AS1411 (acute myeloid leukemia) and NOX-A12 (multiple myeloma and non-Hodgkin’s lymphoma) are evaluated in phase-II and phase-I clinical trials, respectively [20].

In this study, we developed a novel aptamer (HCA#3) that specifically targets HepG2 cells. The binding affinities of HCA#3 to 12 cancer cell lines were recorded by flow cytometry assays, which revealed that aptamer HCA#3 had a high binding affinity for HepG2 and an apparent dissociation constant K_d_ = 305 nM (Fig 2b). The specific binding of the selected HCA#3 to the target cells was further confirmed by fluorescence imaging (Fig 3a). Furthermore, the conjugated aptamer-drug complex (ApDC) was prepared via intercalation of the cytotoxic agent Dox in the DNA of the HCA#3 aptamer (Fig 4c) with four Dox molecules (mol/mol) intercalated in each conjugate aptamer-Dox (ApDC) molecule at GC sites. Importantly, ApDC resulted in selective delivery of Dox into HepG2 cells with significantly lower uptake by SK-HEP-1 cells (Fig 4d). In addition, ApDC retained the efficacy of Dox against HepG2 cells, it exhibited considerably lower toxicity against the control cells (Fig 5), and remained stable in human serum. Our study demonstrated that the HCA#3 ApDC aptamer is a promising candidate for selective targeting of hepatocellular carcinoma cells and specific delivery and release of a high Doc payload. Although the HCA#3 aptamer provides a promising tool for targeted HCC therapy, further studies are still necessary to determine the cell surface target of the HCA#3 aptamer. In addition, further evaluation of the HepG2 aptamer ApDC in animal HCC models is also required before clinical studies in humans are pursued [24].

## Supporting Information

S1 Fig SELEX scheme for aptamer selection. (TIF)

S2 Fig Sequence and secondary structure of HCA#1 and HCA#2. (TIF)

S3 Fig Sequence, secondary structure and affinity of truncated aptamer, HCA#3-S1, and HCA#3-S2, compared with parental aptamer HCA#3. (TIF)
