# Supplementary figures and images for: ssDNA Aptamer Specifically Targets and Selectively Delivers Cytotoxic Drug Doxorubicin to HepG2 Cells
Source: PLoS One. 2016 Jan 25;11(1):e0147674. doi: 10.1371/journal.pone.0147674 (PMC4726709; doi:10.1371/journal.pone.0147674)

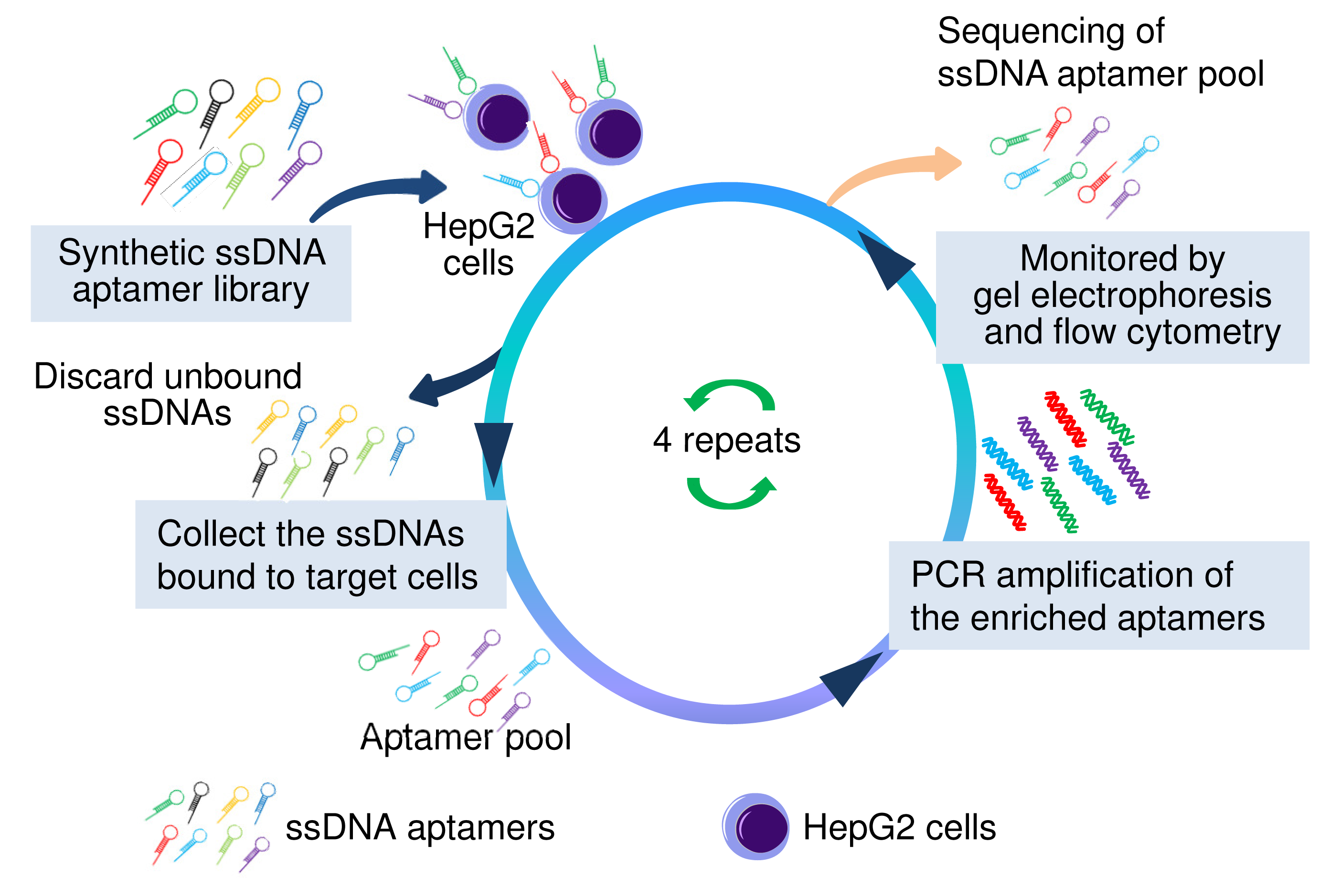

Supplement: S1 Fig — (TIF) [file pone.0147674.s001.tif]

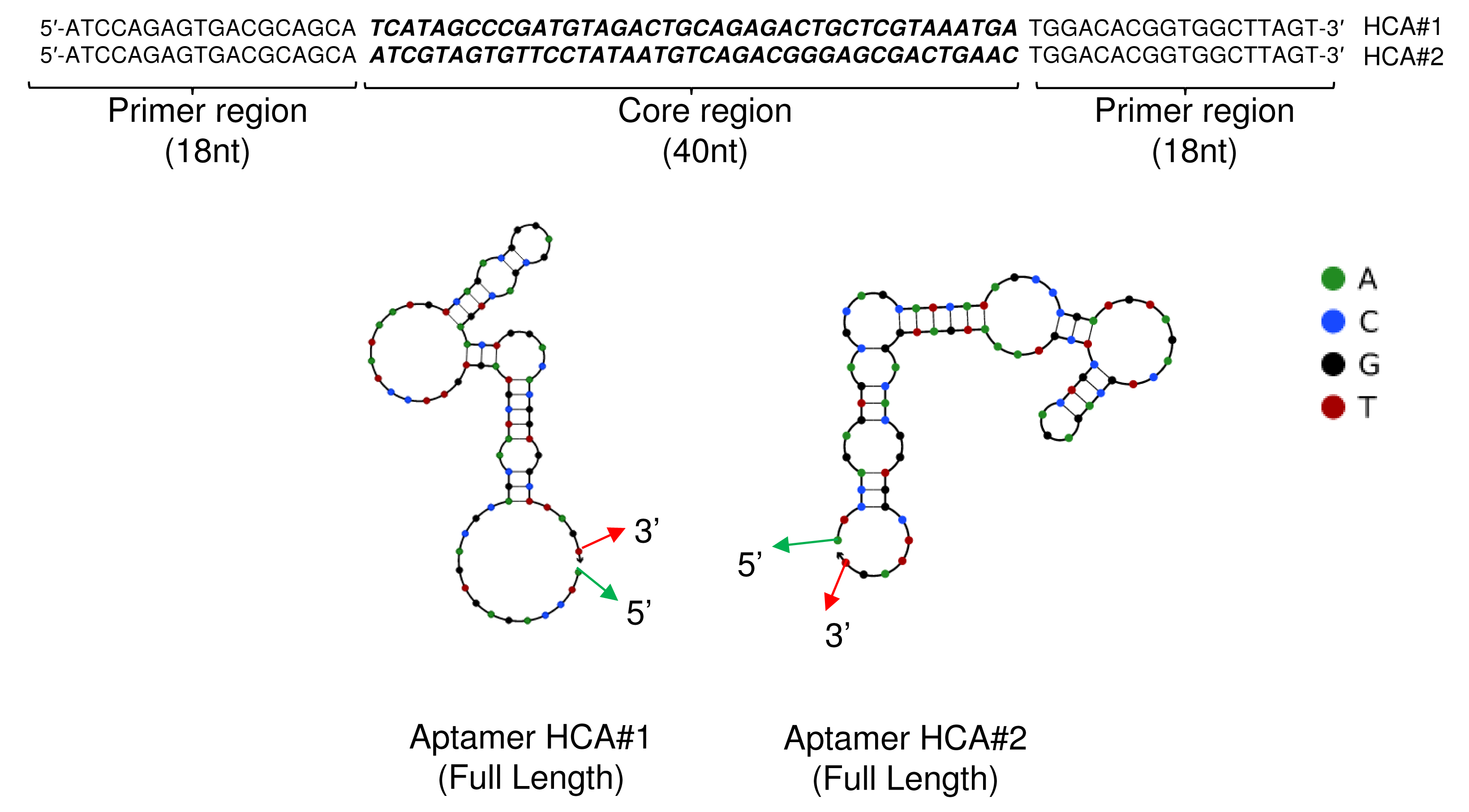

Supplement: S2 Fig — (TIF) [file pone.0147674.s002.tif]

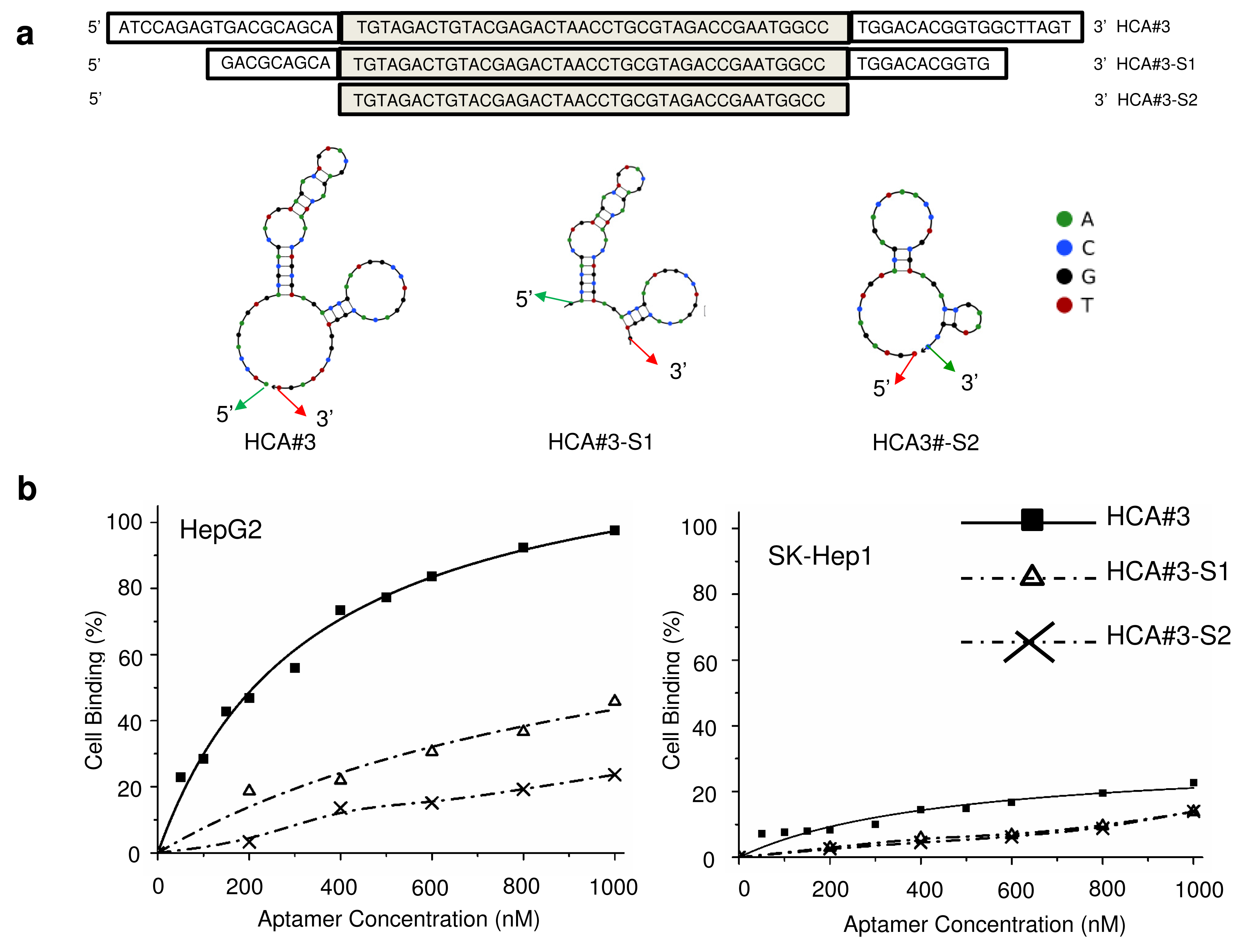

Supplement: S3 Fig — (TIF) [file pone.0147674.s003.tif]
